# *‘*Even though a man takes the major role, he has no right to abuse*’*: future male leaders’ views on gender-based violence in Sri Lanka

**DOI:** 10.1080/16549716.2017.1348692

**Published:** 2017-07-28

**Authors:** Elisabeth Darj, Kumudu Wijewardena, Gunilla Lindmark, Pia Axemo

**Affiliations:** ^a^ Department of Women’s and Children’s Health, International and Maternal Child Health, Uppsala University, Uppsala, Sweden; ^b^ Department of Public Health and Nursing, NTNU, Norwegian University of Science and Technology, Trondheim, Norway; ^c^ Department of Obstetrics and Gynecology, St Olav’s Hospital, Trondheim, Norway; ^d^ Department of Community Medicine Health, University of Sri Jayewardenepura, Colombo, Sri Lanka

**Keywords:** Qualitative, gender roles, men’s perspective, future leaders, Sri Lanka

## Abstract

**Background**: Distinct gender roles influence gender inequality and build the foundation for gender-based violence. Violence against women is a major public health problem in all societies, and a violation of human rights. Prevalence surveys on gender-based violence have been published from Sri Lanka, but qualitative studies on men’s perceptions are lacking.

**Objectives**: The aim of this study was to explore young educated Sri Lankan men’s perceptions of violence against women.

**Methods**: Seven focus-group discussions were held. Men at the end of their university studies were purposefully selected. A topic guide was used, covering various scenarios of violence against women. Qualitative content analysis was carried out.

**Results**: Four categories were developed through the analytic process: fixed gender roles – patriarchal values are accepted in society, female mobility control, and slowly changing attitudes; violence not accepted but still exists – sexual harassment exists everywhere, different laws for different people, female tolerance of violence, and men’s right to punish; multiple factors cause violence – alcohol, violent behavior is inherited, violence culturally accepted, low education, and lack of communication; and prevention of violence against women – both parents must engage and socialize girls and boys equally, life skills education, premarital counselling, working places value clarification, and more women in politics and boards are suggested.

**Conclusions**: Medical and management students, possible future male leaders of the country, have suggestions of prevention strategies in life skills to reduce gender-based violence and to increase knowledge of health consequences with the aim of changing attitudes.

## Background

The distinct gender-related social norms and behaviors influence inequalities between men and women and build the foundation for gender-based violence (GBV). Intimate partner violence is the most frequent form, and efforts continue to be made to find efficient tools for reducing this kind of violence within different cultures and populations. The most effective would appear to be to hinder violence through primary prevention. This would involve early interventions within families, where children follow the parent’s pattern of solving problems, and in schools, where corporal punishment is allowed [1]. Secondary prevention to reduce repeat violence should not only involve perpetrators and behavior-changing programs, but also communities, health workers, the police, and law enforcement to improve awareness and knowledge of the health consequences of violence. Both kinds of prevention refer to the socio-ecologic theoretical model first introduced by Bronfenbrenner [2], and later revised as a modified framework by Heise, of intimate partner violence, explaining the causes of GBV [3]. However, prevention strategies need accurate knowledge of the prevalence, knowledge, and attitudes within the targeted population.

The World Health Organization (WHO) reports that 35% of women worldwide have experienced violence [4]. Nearly a third of ever-partnered women have experienced physical and/or sexual violence by an intimate partner. Most commonly affected are women in South East Asia, which reports a 37.7% prevalence of GBV [4]. In the present study, GBV follows the WHO definition, which refers to any act of GBV that results in physical, sexual, or psychological harm or suffering to women [5].

As in many parts of the world, GBV is a common and major public-health problem and is considered as a serious human-rights violation in Sri Lanka. Sri Lanka has ratified a number of international conventions and laws relevant to GBV, such as the Universal Declaration of Human Rights and the UN Declaration of Elimination of Violence against Women [6,7]. The Sri Lankan government has adopted the Domestic Violence Bill and other policies around GBV [8,9]. The Maternal and Child Health Policy recognizes the role of the health sector in responding to GBV, and the Ministry of Health called for service points to provide care for abused women in 2012. Despite these provisions, violence against women is a common occurrence in many parts of the country. In Sri Lanka, the incidence of GBV and intimate partner violence among ever-married women is estimated to be between 20% and 60% in different studies [10,11].

A cross-sectional study conducted among 1,568 male participants by CARE International in four districts in Sri Lanka focused on males’ attitudes toward gender roles [10]. A majority of men agreed with statements such as: ‘There are times when a woman deserves to be beaten’ and ‘Women’s primary responsibility is taking care of the family and household’. Further, the men connected manhood to dominance and violence, and a majority agreed with the statement, ‘It is manly to defend the honor of your family even by violent means’. About one-fourth of the men considered that women should accept teasing of a sexual nature ‘because it is harmless’ [10]. The same study reveals men’s impunity in the families and communities, and most of the men who had perpetrated forced sexual relations were not afraid of being punished, nor had they experienced any violent repercussion from anybody. Most men said that a woman cannot refuse to have sex with her husband and that it is her responsibility to avoid getting pregnant, but they refrained from violence and forced sex during pregnancy [10]. A recent Sri Lankan study focused on male university students also confirms these attitudes [12]. Prevalence and cross-sectional studies on partner violence have been carried out [5,10,11,13]. However, because prevalence estimates of GBV are highly sensitive to methodological factors, underreporting is a threat to their validity [14]. A qualitative study will provide a complementary picture of the situation in a specific context. Most studies focus on female survivors. However, this qualitative study places an emphasis on Sri Lankan young men’s views on GBV, which, to our knowledge, has not been evaluated before. According to the Prime Minster of Sri Lanka during a meeting attended by all Sri Lankan state universities and the Association of Commonwealth Universities 2016, ‘Universities should take active responsibility for acts of ragging and GBV among students and staff’. As previous studies are quantitative, there is a need to identify what beliefs and attitudes the students have while undergoing their academic studies, as they will encounter colleagues, friends, and patients suffering any form of violence. Young men are future husbands, and university students are future key people and policy makers with decision-making power. The aim of the present study was to investigate male university students’ perceptions of GBV, as it is the young generation who will be the future agents of change and possible role models. Obtaining their views will generate knowledge for future interventions.

## Methods

### Study design

A qualitative study design was used to explore young men’s perceptions of GBV, male dominance, and female subordination, as well as their willingness to engage actively in the prevention of GBV in their future occupations. Three scenarios were presented and related to marital abuse, sexual harassment in the workplace, and women’s strategies to promote their career. The participants were asked to reflect and react to the scenarios and discuss their own attitudes, as well as the possibilities of preventing GBV in their future work. The study used focus-group discussions (FGD), as this was considered most appropriate for this explorative study capturing the common understanding and beliefs of the specific phenomenon of GBV within a selected community of young educated men, rather than their individual experiences. It would not be possible to capture such group discussion in a survey with questionnaires or in-depth interviews. The study participants were asked to share only what they felt comfortable discussing. They were assured that the information provided would only be used for research purposes, the principal investigator would keep the material in a secure place, none of their names would be registered, and their identities would not be revealed.

### Study setting

The study was carried out at a large university campus in Colombo, Sri Lanka. This university was selected for convenience, as one of the co-authors had access to facilities and could provide information to the students about the study. The university has seven faculties and 50 departments. Ten thousand students of both sexes are enrolled, representing Singhalese, Tamils, and Muslims. The majority of the students are residents in the districts close to the campus. Discussions took place in a private room on the campus, which no other students or staff had access to during the FGDs.

### Study participants

Participants were purposefully selected and invited to the FGDs. Inclusion criteria were: male university students from the disciplines of medicine and management studies. They were included if they were in their penultimate or final year of their degree and were starting to prepare for their professional careers. Being married was not an exclusion criterion. Four FGDs with medical students and three FGDs with management students were conducted. In all FGDs, representatives from the different ethnicities and religious communities were included, and the participants were 21–24 years of age.

### Data collection

A topic guide was used during the discussions of the three short scenarios, which were read out loud. One of the authors (KW) moderated the FGDs. She did not teach any of the students. She is experienced in qualitative research, particularly in gender-based research, and has previously worked in projects on masculinity. An additional research assistant observed and took notes duration the sessions, and two of the international co-authors, both with experience of studies on GBV in Sri Lanka, attended four FGDs as observers. The discussions were held in English. They lasted on average 75 minutes (range 60–90 minutes) and were recorded after gaining approval from the participants. All discussions were transcribed verbatim. The number of FGDs was not determined in advance. They continued until the research team found that the material was saturated and rich in information and that no new opinions emerged in the discussions.

### Analysis

The process of analyzing the data was conducted according to qualitative content analysis with systematic text condensation according to Graneheim and Lundman [15]. The transcripts were read several times by all authors, meaning units were identified and condensed without losing the meaning of the original text. Afterwards, the condensed units were coded and merged into subcategories and further into categories. The analytic process was iterative and advanced in progressive cycles continuously as the data were collected. The socio-ecologic theoretical model, with a framework for violence prevention, was used to structure and interpret the findings [3].

## Results

Seven FGDs were held with five to seven students in each group, with a total of 42 participants. Four main categories, each with subcategories, emerged during the analysis (Table 1).

**Table 1.** Categories and subcategories that emerged during the analysis of focus-group discussions among male university students in Sri Lanka

Categories	Subcategories
Fixed gender roles	Patriarchal values accepted in society
	Female mobility control

	Attitudes are slowly changing
Violence not accepted but still exists	Sexual harassment is everywhere
	Different laws for different people
	Female tolerance of violence
	Men’s right to punish
Multiple factors cause violence	Alcohol
	Violent behavior inherited
	Violence culturally accepted
	Low education
	Lack of communication
Prevention of violence against women	Both parents engaged
	Girls and boys equally socialized
	Life skills education
	Premarital counselling
	Workplaces value clarification
	More women in politics

### Fixed gender roles

The students described that patriarchal values are accepted in the hierarchically structured society in which they were raised, with perceived fixed roles within the families and within society. Cultural values and traditions recognize the father as the head of the family, and he makes all the decisions. The wife and children should obey, and after marriage, the woman belongs to the husband. The young men described that a divorce is almost an impossible step for a woman to take. The process is drawn out over many years, and a divorced woman will most probably not be able to marry again bcause of severe social stigma and shame. This was indicated in comments such as ‘In Sri Lanka, most men think that women should be considered inferior to them’; ‘Yes, in Sri Lanka, there is a patriarchal society, so men want to be powerful. To become powerful, he might try to suppress the woman” (Management FGD I); and ‘Even females say males are superior to females, when males cry, they say, “Why are you crying like a woman?” … There are some hidden messages like that’ (Medical FGD II).

Further, in the discussions on gender equality, students indicated:

'A woman should not be given equal status but a suitable status. A man should be the head of the household … The husband has a greater responsibility. If the woman is trying to be superior to her husband, then they should discuss about it without getting violent … the wife should respect the husband.' (Management FGD I)

During the courting period, women have a certain freedom to make decisions for themselves. Many women study and are professionals. However, after marriage, changes are made. It is common according to the discussions that they will leave their jobs to stay at home to care for the home and the children. Opinions among the young men were that if the women continued working after marriage, it would cause difficulties. She cannot take nightshifts at hospitals or work late, as bus transportation is not safe and there is a fear of sexual abuse. Neither can a woman have a top job in business, as they were perceived to be unable to take risks, an undesirable quality for most companies’ employees. Being a teacher was considered to be a more suitable job for a woman, but the young men discussed how women preferred to stay at home or take on employment in the government, which is seen as less demanding. However, if a woman continues her work, according to the participants, the husband is entitled to receive her salary. Women were not supposed to have the ability to reach higher levels of responsibility. This was highlighted in comments such as: ‘There are fewer women in top posts in companies in our country. There is an attitude in society … that it is difficult for women to handle responsibilities’; ‘It is generally believed that men are better at risk-taking than women. Men like to take risks, women are afraid to take risks, and companies look for risk-takers, since risk-taking is involved in making profit’; and ‘When driving vehicles, women are slow at taking decisions, so women are slow at taking decisions and such factors matter. This is why they do not reach high positions’ (Management FGD I).

Despite the inherited hierarchy, students described that the old traditions are slowly changing. Some women, especially those who are educated, are aware of human rights and expect to be treated equally. This was seen to be less common among girls and women living in rural areas, and thus violence is more prevalent there, according to the discussions: ‘But in the cities women are more empowered and have an income, friends … They can be independent; they have a status’ (Management FGD I).

The media play a dual role, as they show modern Western life-styles, which promote women’s liberation, opposing Sri Lankan traditions: ‘If you look at advertisements … it is the male who dresses properly … females are half-dressed. They [the media] are trying to enforce females as objects.’ (Medical FGD I)

The media also play an educational role, enlightening people about women’s rights in order to reduce violence in society, which was an issue discussed in the groups: ‘These types of incidents [violence] are getting lesser now. In our faculty, 2/3 are females. Now they are getting more educated and independent. As women are more educated, they know their rights … and they know that they can speak’ (Medical FGD III); ‘Women in villages were used to tolerating everything. But in the cities women are capable, they have an income, even if they get divorced, they can look after their children. They can be independent, they can have a status’ (Management FGD I).

### Violence not accepted but still exists

A common opinion among the participants in the discussions was that neither GBV nor verbal abuse should be accepted: ‘Even though a man takes the major role he has no right to abuse’; ‘Abusing is not correct. Most of my friends also think the same way what I am thinking. They don’t like to abuse a woman … Physically men are dominant, but mentally man and woman, both are equal. Verbal communication is also the same. Hurting point everything is the same for man and woman’; ‘Most of my friends think the same way. Don’t abuse women’; ‘Husband can’t be like that. My position is … it should not be done. I think she should do something about it. She should talk to him. Go to the police if he has abused too much’ (Medical FGD I).

However, in the FGDs, a man’s right to punish his wife was discussed, for example the attitude that when a conflict arises in a family, the woman should listen to the husband and should not argue, as feelings of anger and frustration may be hard for men to control. If a woman insists on augmenting the disagreement, he will hit her and, from that perspective, both parties are to blame. Simultaneously, this culturally accepted opinion was opposed and support was expressed for making allowances for women to have their own opinion and voice and that they should dare to speak out: ‘Women are restricted more than they should. Women don’t get a proper place because the parents restrict them. So they should be aware not to do so’ (Management FGD I).

There were also options that women had responsibility not to provoke men: ‘The woman should make sure not to start a quarrel’ (Management FGD II).

However, sexual harassment is prevalent everywhere, including the workplace, and superiors can misuse their positions. The students described that if they witnessed a female colleague or a nurse being harassed by someone higher in the hierarchy, they felt that they have very limited options to react because of fear of not being approved in an exam or losing their academic standing. Possible solutions included suggesting that they talk to the girl or a friend, but formal actions would be hard to take. The management students believed that companies had more structural arrangements that could be used in these situations: ‘The person who speaks out will be in trouble the next morning’ (Medical FGD II); ‘In most companies, there is a HR division, disciplinary acts can be taken’ (Management FGD IV).

The medical students claimed that once they have graduated and work professionally, they will be more independent and can act in favor of the harassed women: ‘I will stay until I get the degree, then I will go ahead and complain about the harassment’; ‘If you are a superior, the juniors tend to be, you know, very down and out … they will never stand up to a senior officer’ (Medical FGD II).

In addition to observing abuse of nurses in hospital wards, the students also observed women they provided care for being violated, and by nurses who disclosed and laughed at the patient’s situation. There seems to be few opportunities to provide counseling to the injured women at the hospital. This was all seen as a silent acceptance of the existing violence: ‘In our country, women are not aware of the help they can get from the institutions … she doesn’t have any legal knowledge’ (Medical FGD III).

The young men also discussed how the young women used their femininity to achieve advantages, such as higher grades or improved academic standing: ‘Some females use their femininity to … get some things. Like getting phones or tickets’; ‘It happens especially in exam situations. When presenting a case, the consultant will definitely give marks. He will fail girls who don’t like him’ (Medical FGD III).

When the participants discussed what actions they would take if they saw a colleague being abused, they indicated that they would not take direct action, but that they would try to take action indirectly, as they were afraid of the consequences. However, if it was their own sister who was being abused, their responses were different: ‘Family comes first’ (Medicine FGD III).

Still afraid of losing a job or not passing an exam, the participants suggested that an anonymous letter could be sent to the head of the company to ‘bite the boss’ if he was the perpetrator. The students agreed that there were different consequences for abuse according to social status. They were aware of the laws prohibiting violence. However, thoughts were articulated that superiors had the possibility to influence the authorities. Members of parliament, politicians, and the policecould be manipulated or might even be offenders themselves, and the laws did not always seem to be adhered to: ‘There are different laws for different people. They make the law, and they break the law’ (Medical FGD I).

In spite of the existing laws, women often do not report domestic violence in order to protect their families’ reputation. This makes it hard to influence change when violence continues in families. Mothers are seen to support this culture and bring up their children to behave in a similar way. An opinion in the discussions was that women had the responsibility to end quarrels and to be patient, as the men might be under pressure, which may lead to conflict. Alternatively, men have the responsibility to control their wives, and suspicions of female adultery were commonly discussed. However, the young men’s understanding was that women are accustomed to having to tolerate violence and the men’s control of their mobility.

### Multiple factors cause violence

Alcohol use and alcoholism were seen, in all discussions, as major contributions to domestic violence and sexual abuse: ‘I think culture, ignorance, alcoholism … cause this problem’; ‘Alcoholism, teenage marriage, and our culture are the reasons for abuse’ (Medical FGD II); ‘He may be an alcoholic or having an affair with another woman’ (Management FGD II).

Low income and frustration combined with low education and living in rural areas were perceived as risk factors for using violence to solve conflicts. As this violent behavior may be socially accepted, it can end up in a negative circle and increase the risk of repeating violence against women in the new generation. Even if the woman is admitted to hospital with obvious injuries caused by her husband, she may return home to him, a situation that is culturally accepted, as there is not enough social support and no economic support system in Sri Lanka to care for abused women. If the husband is jailed because of his abuse, the shame still falls on the woman: ‘In villages, if a man is hitting his wife and he is put in jail, then the woman will think it is an embarrassment to her. She will think like that’ (Management FGD III).

Dress codes and places were discussed, among both the medical and management students, as violating the accepted dress code in the workplace or being in the wrong environment could provoke violent behavior: ‘If a girl is wearing a decent frock, and there is another girl wearing a short frock … any boy would look at the girl in the revealing outfit. Dress is a main reason, even for rape’; ‘No one would look twice at a covered woman’; ‘Yes, dress plays a role … if they show their body parts to everyone, everyone will want to touch them” (Management FGD II)

'The girl and the place play a role. In parties with well-mannered people, the dress will not matter. In a bus station or on a train station, if she wears the same dress … there will be people who are uneducated or drunken … it would provoke them, and there would be some abuse.' (Medical FGD I)

The participants described how violence is facilitated when communication is lacking in a married couple’s relationship. After an arranged marriage, the husband and wife do not know each other, and the expected subordination of the woman makes it harder for both of them to discuss a problem. Schools are often not of mixed gender, the roles are predetermined, and skills in how to communicate with the opposite gender are not developed. Subsequently, violence is used as a way to solve conflicts instead of verbal communication: ‘Throughout their lives, they should have good interaction. Then only we could learn about each other’ (Medical FGD II).

'You got married without knowing the person properly, like arranged marriages … so you don’t actually knew the person until you … are married and signed on the dotted line. Then you find the discrepancies and … the differences arise. Whereas in another country, you live with each other, and you decide you are compatible with each other and then get married. There, women can leave anytime … but in Sri Lanka, when you get married, there is a legal lock-down and you are there forever.' (Medical FGD III)

### Prevention of violence against women

Perceptions expressed in the FGDs were that girls and boys should be socialized equally and that admission to mixed schools should be encouraged. Loving parents should have mutual respect and be engaged in the children’s upbringing. Equal treatment for boys and girls was recommended. Adults should adhere to human values and oppose GBV. In order to achieve these changes, premarital counselling should be available for couples, and children should be introduced to life skills education early, even at preschool level, and this should continue in school, supported by parents and religious leaders.

The participants had lively discussions about how they could intervene when violence had occurred in the workplace. As individuals, they felt restricted, but thoughts of going together as a group to the management and informing them of violent incidents or sexual harassment would be a safer and more appropriate option. Further, they suggested that in providing care for GBV, victims will need interdisciplinary teams of physicians, social-care partners, and legal authorities. Raising awareness within the community was perceived as important, and examples, such as distributing posters and stickers relating to the impact of violence, were seen as valuable attempts to reduce violence. The young men wanted more women to engage in politics and governmental support and follow up already existing declarations on eliminating violence against women: ‘Girls and boys should interact. Here should be mixed schools’; ‘When girls get menarche, the girls are put into a room and not supposed to see any boys’; ‘That sends very negative messages to a young girl’s mind’ (Medical FGD I); ‘We can start sharing the duties at home and the children will see’ (Medical FGD II); ‘In Sri Lanka, counseling for families doesn’t take place. I think it should happen’ (Management FGD II). ‘Schoolchildren should be aware of this [violence] … educated on how to lead a family life, about society, about the way they should live’; ‘…and how to treat women’ ‘Personality development is very important. Girls should be educated about this. They should be strong to stand up against harassment … Women in parliament should be increased. Even the Minister of Women’s Affairs was a man, which should not happen’ (Management FGD IV).

## Discussion

This study reveals that the male university students see themselves as being at a crossroads. Patriarchal hierarchy and normative masculine and feminine roles are still strongly prevalent, and they see the man not only as the decision maker and head of the family, but also when it comes to controlling women. At the same time, they are aware of the conflict between the patriarchal values of the society and the liberal, egalitarian attitudes toward women,and they adhere to the view that GBV should not be accepted within society. The students presented various suggestions in order to reduce and prevent violence, such as less alcohol consumption, education on gender and violence prevention at an early age, marital counseling, and law enforcements. Other studies from Sri Lanka have focused more on men in general or on participants of both sexes. We wished to understand the thoughts of the future male leaders, a highly educated group. The present study adds to results from a Sri Lankan quantitative study performed 10 years ago, where most students, both men and women, justified wife beating, claiming that woman was responsible for the violence she suffered [10,16]. We could not confirm these opinions during our discussions, and after 10 years, it might be less sensitive to talk about GBV and discuss traditions and various opinions, as advocacy programs have been organized and referred to in the media. We found complex and detailed information about the students’ perceptions and experiences on the transition from traditional patriarchal beliefs to a new view of women’s equal rights, both in private and in the workplace. Thus, a rethinking of traditional values seems to be occurring, but this is also anchored in the culture, even if on a rational level they are aware of women’s rights and the necessity for themselves to establish a new male role model.

The strength of this study is the qualitative approach, as, to our knowledge, none has been performed previously in Sri Lanka on this topic. These young men, who were recruited from the health and management sectors, will have the power to influence the attitudes of their employers and co-workers in the future. In order to certify trustworthiness and ensure transferability, we have described the study setting, design, method, data collection, analytic process, and participating respondents for the readers. This study was performed by an international team of medical doctors, all with experience in qualitative research methods and studies of the health consequences of violence in developing regions, which increases the credibility of the study.

A possible limitation could be that the moderator was a professor at the university. However, none of the students had met her before or had attended any of her lectures. The moderator, observers, and most of the note-takers were women. However, the students were recruited by a male public-health staff member. The moderator was very careful not to express personal opinions or to be critical of any of the students. They may have been eager to inform the female researchers of the male perspectives or to suppress their thoughts and give socially accepted answers. The impression during the FGDs was that they discussed openly without obvious hesitation.

Our results relating to the perceptions of a willingness to diminish inequality are seen from the perspective of the socio-ecologic theoretical framework at four different levels [3]. At the individual level, the need for education is apparent in order to discuss and change the strictly defined gender roles in society, findings similar to those also supported in a large quantitative survey, the CARE study, previously carried out in Sri Lanka [10]. It is also important that each individual has knowledge of and is aware of their own human rights and that they incorporate a non-accepting attitude to violence in order to reduce the risk of exposure to violence. This is supported by a recent Tanzanian study illustrating poor knowledge and accepting attitudes to GBV [17]. Men with experience of sexual, physical, or emotional abuse during childhood are more likely to execute GBV in adulthood. That is why it is important to visualize the vulnerability of boys to sexual abuse, but also to study the impact it has on the process by which boys who become violent men often target women. An understanding of how stigma, shame, somatic and mental-health consequences, and suicide and homicide due to abuse affect women and strategies to prevent these occurrences should be include in the education of both sexes [18,19].

A lack of communication within the couple was perceived as a major reason for violence at the relationship level. Arranged marriages, which are common in Sri Lanka, can be seen as both positive and negative traditions [20]. Positively relying on parents’ skill in choosing a suitable and matching partner in order to reduce the risk of conflicts may be comforting. On the other hand, not being able to choose a partner for a ‘love marriage’ could cause negative feelings and frustration, leading to the misuse of alcohol and violence within the family. Harmful use of alcohol has been studied and is linked to interpersonal violence, affecting physical and cognitive functions, findings that are in agreement with our results [21–23]. Exposure or being a witness to family violence during childhood increases post-traumatic stress symptoms, psychological adjustment, and use of violence in personal relations [24]. The findings in our study that women should accept their gender role, take care of the children and the household chores, and support men in being tough and family heads encourage the static masculine/feminine roles and are in agreement with a previous Sri Lankan study [10]. However, the young men in this study also suggested that these kinds of attitudes are changing because of an increase in the number of educated women.

At a community level, awareness of GBV and its consequences must be raised, and the establishment of value systems in schools, hospitals, and workplaces was seen as important. Likewise, it was seen as important that laws are adhered to equally by all. Community intervention programs have been introduced with various results, and their long-term effect has been questioned. More creative initiatives need to be explored with the aim of improving secondary prevention of violence. Various shelter programs for counseling and to consolidate the security of women have been established, such as the One Stop crisis centers, Mithuru Piyasa in Sri Lanka, and women’s police stations [25]. Community mobilization, through awareness programs and campaigns, has been launched and addresses different types of violence [25]. A recent study presents the essential engagement of men and boys in intervention action to prevent violence against women and reduce gender inequities. This illustrates the conceptual shift from treating men as perpetrators of GBV to an approach that seeks to transform the relations, social norms, and systems that sustain gender inequality and violence [26]. Successful programs to reduce GBV engage multiple stakeholders in a variety of approaches to address the multiple factors underlying violent behavior.

At the governmental level, laws are already declared and known to these future leaders. However, it is perceived that they are not always followed correctly. Laws against marital rape, which are in place in some countries, were not discussed among the future leaders in the present study. The media were seen to have a specific responsibility to inform general societal attitudes against violence and to reject and fuel old harmful traditions. A promising model has been described by Proosman on home-visiting interventions, which seem to be effective in reducing intimate partner violence [27].

## Conclusions

Male university students in Sri Lanka are aware of the conflict between the traditionally patriarchal values of their society and the modern views of equal rights for women that confront them through the media and in their education. They generally do not accept violence and abuse of women, but they believe that there are social factors leading to acceptance of abuse and preventing social support to the victims. Equal education opportunities for boys and girls and better premarital counselling was recommended, as well as community programs for increased awareness and support for victims of violence.

## Paper context

Patriarchal traditions contribute to and support acceptance of violence and abuse of women, as previous quantitative studies in Sri Lanka have confirmed. Our qualitative study reports attitudes and perceptions among male university students who in their professions will influence future change. Although they generally agree with traditional expectations of gender roles, they also condemn violence and abuse of women. They propose shared education and community mobilization for prevention, and they expect to intervene as professionals.
